# Characteristics, clinical outcomes and patient-reported outcomes of patients with ulcerative colitis receiving tofacitinib: a real-world survey in the United States and five European countries

**DOI:** 10.1186/s12876-023-02640-7

**Published:** 2023-01-19

**Authors:** Alessandro Armuzzi, Ailsa Hart, Joseph C. Cappelleri, Nadir Mammar, Peter Hur, Benjamin Hoskin, Fritha Hennessy, Gary Milligan, Axel Dignass

**Affiliations:** 1 IBD Center, IRCCS Humanitas Research Hospital, Rozzano, Milan, Italy; 2 Department of Biomedical Sciences, Humanitas University, Pieve Emanuele, Milan, Italy; 3 IBD Unit, St. Mark’s Hospital, London, UK; 4 Department of Medicine I, Agaplesion Markus Hospital, Frankfurt/Main, Germany; 5 Pfizer Inc, Groton, CT USA; 6 Pfizer France, Paris, France; 7 Pfizer Inc, New York, NY USA; 8 Adelphi Real World, Bollington, UK

**Keywords:** Ulcerative colitis, IBD, Disease burden, Tofacitinib, Treatment outcomes, Real-world, Survey, Biologics, EU, United States

## Abstract

**Background:**

To describe variations in treatment patterns, clinical outcomes, patient-reported outcomes (PRO), and physician and patient satisfaction in patients with moderate-to-severe ulcerative colitis (UC) treated with tofacitinib in a real-world setting.

**Methods:**

Data were drawn from the Adelphi UC Disease Specific Programme™, a point-in-time survey of physicians and their consulting patients in the US and Europe. For inclusion in this analysis, gastroenterologists completed medical record forms for the next seven consecutive consulting patients with confirmed UC, plus a further two patient record forms for patients treated with tofacitinib. Those same patients then completed a patient-reported questionnaire.

**Results:**

Gastroenterologists (n = 340) provided data for 2049 patients with UC, including 642 patients receiving tofacitinib. Physicians’ most frequent reason for choosing tofacitinib was overall efficacy (71.3% of patients). The proportion of patients in remission increased with length of treatment, from 13.7% at *[0, 4)* weeks to 68.3% at *[52*+*]* weeks. Both physicians and patients reported that the Mayo components of stool frequency and blood in stool were reduced with time on treatment. Improvement in symptoms (bloody diarrhea, abdominal pain/cramps, urgency, rectal bleeding, fatigue/tiredness) was reported in the first weeks of treatment, and increased with time. At week *[52*+*]*, mean score reductions from treatment initiation to current in overall symptom severity, pain, and fatigue were 2.2 (to a current mean score of 1.1), 2.2 (to 0.9), and 2.1 (to 1.0), respectively. Comparing patients at weeks *[0, 4)* and *[52*+*]* (all PROs, *p* < 0.0001), the increase in EQ-5D-5L index total score was 0.29 points and in SIBDQ total score was 20.5 points; percent reductions in WPAI absenteeism was 34.4%, presenteeism 26.8%, overall work impairment 40.9% and activity impairment was 28.3%. These changes reached the thresholds for minimally clinically important differences. The majority of physicians (91.9%) and patients (93.5%) were satisfied with tofacitinib at week *[52*+*]*.

**Conclusion:**

Patients with moderate-to-severe UC treated with tofacitinib show considerable improvement in symptoms and quality of life from tofacitinib initiation to one year and beyond, with high rates of remission. Physicians and patients report satisfaction with UC control at recommended doses in a mostly biologic experienced population.

**Supplementary Information:**

The online version contains supplementary material available at 10.1186/s12876-023-02640-7.

## Background

Ulcerative colitis (UC) is a chronic inflammatory bowel disease, with a prevalence of 286 per 100,000 in the USA, and varying across Europe (per 100,000: Northern 91–505; Southern 15–134; Eastern 2–340; and Western 43–412) [1].

UC is characterized by mucosal inflammation that starts in the rectum and extends proximally for a variable distance through the colon [2, 3]. At presentation, proctitis is most common, found in 30–60% of patients, while 16–45% have left-sided colitis, and 15–35% have extensive disease [4]. Symptoms vary based on severity and extent of disease. UC most commonly presents with blood in the stool (reported by 90% of patients) and diarrhoea [4]. The majority of patients with UC have mild-to-moderate disease [5]. About one-third of patients with UC have extraintestinal manifestations of which spondyloarthritis appears to be the most common [4]. As a consequence, UC has a significant negative impact on many aspects of patients’ quality of life (QoL) [6].

The main intermediate and long-term goal of pharmacotherapy in UC is sustained corticosteroid-free remission, in terms of symptoms and mucosal healing on endoscopy [2, 7, 8]. Guidelines recommend a variety of different therapy classes, including aminosalicylates, corticosteroids, immunomodulators, tumour necrosis factor inhibitors (TNFi), the anti-integrin vedolizumab, interleukin (IL)-12/23 antagonist ustekinumab, and the Janus kinase (JAK) inhibitor tofacitinib, for the induction and maintenance of remission in moderate-to-severe UC [2, 9, 10]. Recently, other JAK inhibitors, filgotinib and upadacitinib, and the sphingosine-1-phosphate (S1P) receptor modulator ozanimod have been approved for the treatment of moderate-to-severe UC. An increasing number of available therapeutic options has led to considerable practice variability in the use of these drugs to treat patients with moderate-to-severe UC [11]. Despite advances in the treatment of UC, clinical and endoscopic endpoints cannot be achieved in a substantial proportion of patients with biologics [12, 13]. Varying persistence profiles [14, 15] suggest that cycling across multiple biologics and novel treatments with different modes of action are warranted.

Tofacitinib is an oral, small molecule, JAK inhibitor for the treatment of moderate-to-severe UC that was approved in Europe and the USA in 2018. Tofacitinib has demonstrated effectiveness including remission, mucosal healing, and patient-reported outcomes in three randomized controlled trials (RCT) [16–18]. However, RCTs are considered to be ‘gold standard’ regarding evidence-based medicine, but their strict patient inclusion/exclusion criteria means that they do not necessarily reflect real-world patient populations, thus limiting their generalizability. Real-world evidence is therefore important in demonstrating effectiveness during routine clinical practice, where conditions are not so tightly controlled. Physicians and patients can provide unique and independent assessments of disease activity, which can be used in treatment decision making. Therefore, to support the robust tofacitinib clinical trial data, we present data from the well-established Adelphi Ulcerative Colitis Disease Specific Programme™ (DSP) [19]. The objective of this analysis was to describe the variations in treatment patterns, patient-reported outcomes and physician and patient satisfaction, in patients with moderate-to-severe UC treated with tofacitinib in a real-world setting.

## Methods

### Survey design

Data were drawn from the Adelphi Ulcerative Colitis Disease Specific Programme™ (DSP) [19], a large, multinational, point-in-time survey of physicians and their patients. The survey comprised a physician survey, with medical record data abstraction by physicians, and a patient survey. Data were collected from Q3 2020 to Q1 2021 from the USA and five European countries, including France, Germany, Italy, Spain, and the UK. The DSP methodology has been previously published and validated [19–21]. The DSP was conducted in accordance with the Western Institutional Review Board (protocol number A3921382), and required informed consent from physicians and patients before their participation.

### Participant selection and data collection

Physicians (gastroenterologists) were eligible to participate in the DSP survey if they were personally responsible for and actively involved in treatment decisions and management of patients with UC, and had a clinical workload of 7 or more patients with UC in a typical month.

Patients were eligible for inclusion in the DSP survey if they aged 18 years or older, had a physician-confirmed diagnosis of UC, and visited the physician. Patients who were involved in a clinical trial were excluded.

Patients included in the data analysis were a combination of a subset of these randomly sampled patients (the main sample), and a deliberately captured additional set of patients (the over-sample). The main-sample patients were those captured by physicians during random sampling. The over-sample only included all patients (regardless of current disease severity) with UC prescribed tofacitinib. All patients in the analysis were those who were considered to have moderate-to severe disease, as defined by patients who had received immunomodulators (as monotherapy or in combination with corticosteroids), biologics, biosimilars or JAK inhibitors at some point in their treatment journey.

Gastroenterologists completed a patient record form for their next 7 consecutive patients (main sample) with UC who visited their office for routine care. This physician-reported questionnaire form contained questions on demographics, clinical characteristics, treatment history and pattern, and treatment satisfaction. Gastroenterologists completed the patient record form through consultation of existing patient clinical records and, consistent with decisions made in routine clinical practice, their judgement and diagnostic skills. They then provided data for the tofacitinib oversample by completing up to a further two patient record forms on a prospective basis.

Physicians then invited the patients for whom they completed a patient record form to complete a patient-reported form. The patient-reported form collected data on stools, treatment satisfaction, and health-related QoL (HRQoL). The patient-reported form collected HRQoL data on the emotional and physical impact of UC using the EuroQol- 5 Dimension-5 Level (EQ-5D-5L) index [22] and the short version of the Inflammatory Bowel Disease Questionnaire (SIBDQ) [23, 24], and the impact of the condition on daily functioning using the Work Productivity and Activity Impairment (WPAI) questionnaire, specific health problem (WPAI-SHP; i.e., ulcerative colitis) version [25, 26].

This analysis used coded data that already existed in a secondary electronic database. A survey number was assigned to all participating physicians and patients to enable anonymous data collection and data linkage during data collection and analysis. This enabled patients’ responses to be matched with their corresponding physicians’ responses, and thus evaluate whether patients and their gastroenterologists were aligned in their perceptions of aspects of disease severity. Responses were therefore anonymized before aggregated reporting, the identity of the physicians was blinded, and no patient identifiers were collected.

### Study measures

Study measures, along with their associated score ranges and minimal clinically important differences (MCID) are shown in Table 1. Included are the EQ-5D-5L index that evaluates health status/HRQoL in terms of mobility, self-care, usual activities, pain/discomfort, and anxiety/depression. Health state index scores generally range from less than 0 (where 0 is the value of a health state equivalent to dead; negative values representing values as worse than dead) to 1 (the value of full health). A 0.074-point change in the EQ-5D scale is considered a minimal clinically important difference (MCID) [22, 27]. The SIBDQ total score that assesses HRQoL in terms of social, emotional, and physical well-being on a scale ranging from 10 indicating worst health to 70 indicating best health is also included. A 9-point change in the SIBDQ is considered the MCID [23, 24]. Lastly, the WPAI (disease specific for UC) that measures UC-related time missed from work, and impairment of work and regular activities is also included; greater values indicate more UC-related impairment caused by the WPAI component. The WPAI component scores are reported as percentage impairment. There are no published MCIDs available for the WPAI in UC; therefore, we used the thresholds reported for Crohn’s Disease that have been estimated to be 6.5% for absenteeism, 6.1% for presenteeism, 7.3% for overall work impairment, and 8.5% for total activity impairment [26, 28, 29]. Disease activity of UC was assessed using the partial Mayo score [30–32], and overall symptom severity, overall pain severity, and overall severity of fatigue/tiredness were rated by physicians.

**Table 1:** Study measures

Measure	Area of evaluation	Score range	MCID
EQ-5D-5L index	Health status/HRQoL	Total score ranges from 0.00, worst health/HRQoL to 1.00, best health/HRQoL	0.074-point change [27]
SIBDQ total score (patient rated)	HRQoL	Scale ranging from 10, worst health to 70, best health	9-point change [24]
WPAI (UC adapted) (patient rated)*	Absenteeism, presenteeism, overall productivity impairment, and impairment of regular activities	Scale ranging from 0%, no impairment to 100%, total loss of work productivity or activity	6.5% for absenteeism; 6.1% for presenteeism; 7.3% for overall work impairment; and 8.5% for total activity impairment [28]
Partial Mayo score (physician rated)	Disease activity (stool frequency, rectal bleeding, and physician’s global assessment)	Scores: < 2, remission; 2–4, mild activity; 5–7, moderate activity, and > 7, severe activity	N/A
Overall symptom severity Overall pain severity Overall severity of fatigue/tiredness (all physician rated)	Overall symptom severity, overall pain severity, and overall severity of fatigue/tiredness	Scale ranging from 0, no symptom to 5, extremely severe symptom	N/A

EQ-5D-5L, EuroQol- 5 Dimension-5 Level index; HRQoL, health-related quality of life; MCID, minimal clinically important difference; SIBDQ, short version of the Inflammatory Bowel Disease; UC, ulcerative colitis; WPAI, Work Productivity and Activity Impairment questionnaire

*There are no published MCIDs available for the WPAI in UC; therefore, we used the thresholds reported for Crohn’s Disease

### Statistical analysis

The main analyses were conducted on patients who had been treated with tofacitinib, excluding the 10 patients who were initiated on tofacitinib on the day of consultation or whose date of tofacitinib initiation was unknown. Unless otherwise stated, data reported are at the time of data collection (i.e., each patient contributes one observation concerning their “current” state). Primary endpoints were demographics, treatment pathways prior to the initiation of tofacitinib and symptomatology. Secondary endpoints were tofacitinib dosing, steroid patterns and clinical response to tofacitinib.

For demographic and clinical characteristics, patients were divided into three groups by biologic treatment, whereby tofacitinib had been administered to patients who were biologic naïve, had received treatment with a single biologic, or had received treatment with ≥ 2 biologics. Biologic therapy in this analysis comprised immunotherapy and targeted therapies (TNF-alpha inhibitors, anti-integrins, and IL12/23 inhibitors).

Data were summarized using descriptive analyses. Means and standard deviations (SD) were calculated for continuous variables (number of observations), and frequency and percentages were calculated for categorical variables (patient numbers). Missing data were not imputed and therefore, the base of patients for analysis could vary from variable to variable and is reported separately for each analysis.

For analysis of treatment patterns and clinical outcomes, tofacitinib patients were divided into groups according to week of tofacitinib treatment, specifically weeks *[0, 4), [4, 8), [8, 16), [16, 24), [24, 52), and [52*+*]*. Comparisons were made between the treatment groups.

Standard statistical analyses were performed [33]. Specifically, patient characteristics were compared using univariate tests (analysis of variance for continuous outcomes and chi-squared test for categorical outcomes). Patient outcomes (e.g., EQ-5D, SIBDQ, and WPAI) were compared using regression (linear for continuous, logistic for categorical outcomes) that included covariates age, sex, body mass index, Charlson comorbidity index, and number of previous advanced treatments. Least square means (with n and 95% confidence intervals) were reported within the treatment periods. For each regression, a *p*-value was calculated that tested the joint hypothesis that the coefficients for all the treatment periods were zero using Wald tests, that is the treatment periods are not statistically associated with the outcome. Note that patients contributed one observation each, which means that different time periods were composed of different (and independent) groups of patients.

## Results

### Physician-reported patient demographics

For this analysis, 251 gastroenterologists provided data for a total of 652 eligible patients with UC receiving a JAK inhibitor (i.e., tofacitinib); 83 from main sample and 569 from the oversample. A total of 164 eligible matched patients provided their data.

**Fig. 1:**
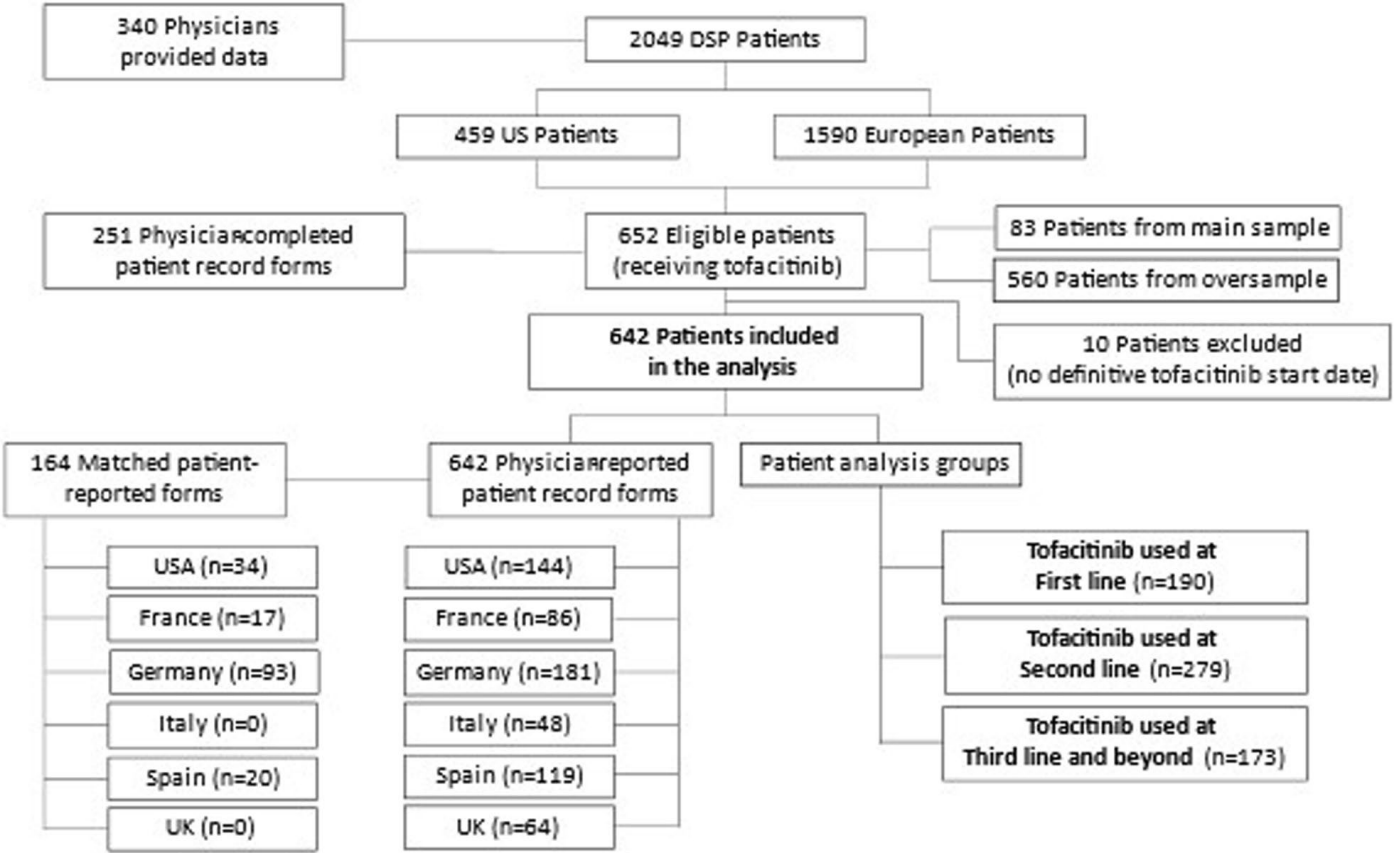
Selection pathway of patients with UC in the analysis groups. Legend: Patients were abstracted from the patient data collected in Adelphi Ulcerative Colitis Disease Specific Programme™ (DSP) survey

As part of the total evaluated Adelphi UC DSP sample, 340 gastroenterologists provided information for a total of 2049 patients with UC; 459 patients from the USA and 1590 from the five European countries (Fig. 1).

Physicians completed a patient report form for 652 patients with UC receiving a tofacitinib, from the USA (n = 149, 22.9%), France (n = 87, 13.3%), Germany (n = 182, 27.9%), Italy (n = 48, 7.4%), Spain (n = 119, 18.3%), and the UK (n = 67, 10.3%). Ten patients either had no definite start date for tofacitinib or were initiated on tofacitinib on the day of data collection, and were excluded from further analysis.

Patient-reported forms were completed by 164 matched patients from the USA (n = 34, 20.7%), France (n = 17, 10.4%), Germany (n = 93, 56.7%), Italy (n = 0), Spain (n = 20, 12.2%), and the UK (n = 0).

For subsequent analysis, 642 patients with UC were grouped according to weeks of tofacitinib treatment, as follows: *[0, 4)* weeks, n = 39, 6.1%; *[4, 8)* weeks, n = 48, 7.5%; *[8, 16)* weeks, n = 112, 17.4%; *[16, 24)* weeks, n = 76, 11.8%; *[24, 52*+*)* weeks, n = 201, 31.3%; and *[52*+*)* weeks, n = 166, 25.9%.

### Physician-reported patient clinical profile

Patient characteristics and clinical profile are shown in Table 2; n = 642. Of 642 patients with UC, 190 patients (29.6%) were biologic therapy naïve (i.e., received tofacitinib as their first advanced therapy and had never received a biologic), 279 (43.5%) were initiated on tofacitinib after previous exposure to a single biologic therapy, and 173 (26.9%) were initiated on tofacitinib after previous exposure to ≥ 2 biologic therapies, respectively.

**Table 2:** Demographic and clinical characteristics of patients with moderate-to-severe UC receiving tofacitinib at data collection

Characteristic	Total (N = 642)	Biologic naïve (N = 190)	Post 1 biologic (N = 279)	Post ≥ 2 biologics (N = 173)^a^
Time from diagnosis to tofacitinib initiation, years				
Missing, n	71	26	25	20
Mean (SD)	4.7 (5.5)	1.4 (2.2)	5.2 (5.4)	7.6 (6.2)
Age at initiation of tofacitinib				
Mean (SD)	38.5 (11.3)	37.3 (12.0)	37.4 (9.6)	41.7 (12.3)
Current age, years, n (%)				
Mean (SD)	39.3 (11.3)	38.0 (12.2)	38.2 (9.5)	42.4 (12.3)
≤ 50	540 (81.4)	167 (87.9)	246 (88.2)	127 (73.4)
≥ 50)	102 (15.9)	236 (12.1)	33 (11.8)	46 (26.6)
Sex, male, n%				
Male	364 (56.7)	111 (58.4)	149 (53.4)	104 (60.1)
Ethnicity, n%				
White/Caucasian	571 (88.9)	163 (85.8)	254 (91.0)	154 (89.0)
Hispanic/Latino	22 (3.4)	7 (3.7)	7 (2.5)	8 (4.6)
African American	12 (1.9)	9 (4.7)	3 (1.1)	0 (0.0)
Other	37 (5.8)	11 (5.8)	15 (5.4)	11 (6.4)
Current smoking status, n (%)				
Current smoker	58 (9.0)	26 (13.7)	21 (7.5)	11 (6.4)
Ex-smoker	199 (31.0)	57 (30.0)	76 (27.2)	66 (38.2)
Never smoked	354 (55.1)	98 (51.6)	164 (58.8)	92 (53.2)
Don’t know	31 (4.8)	9 (4.7)	18 (6.5)	4 (2.3)
Current disease location, n%				
Missing, n	72	22	43	7
Ulcerative proctitis	46 (8.1)	21 (12.5)	12 (5.1)	13 (7.8)
Proctosigmoiditis	102 (17.9)	45 (26.8)	41 (17.4)	16 (9.6)
Left-sided colitis	188 (33.0)	66 (39.3)	83 (35.2)	39 (23.5)
Extensive colitis	234 (41.1)	36 (21.4)	100 (42.4)	98 (59.0)
Current extraintestinal manifestations^c^, n (%)				
Missing, n	16	9	6	1
Yes	100 (16.0)	18 (9.9)	33 (12.1)	49 (28.5)
Current comorbidities (total > 5%), n%				
Anxiety	88 (13.7)	26 (13.7)	29 (10.4)	33 (19.1)
Depression	45 (7.0)	7 (3.7)	17 (6.1)	21 (12.1)
Hypertension	63 (9.8)	24 (12.6)	17 (6.1)	22 (12.7)
Other	200 (31.2)	59 (30.6)	63 (22.)	76 (43.7)
Most frequent UC symptoms at tofacitinib initiation, n%				
Missing, n	8	0	5	3
Diarrhea, bloody	485 (76.5)	128 (67.4)	219 (79.9)	138 (81.2)
Abdominal pain/cramps	486 (76.7)	139 (73.2)	210 (76.6)	137 (80.6)
Bowel movement urgency	454 (71.6)	123 (64.7)	200 (73.0)	131 (77.1)
Rectal bleeding	335 (52.8)	86 (45.3)	149 (54.4)	100 (58.8)
Fatigue/tiredness	292 (46.1)	72 (37.9)	129 (47.1)	91 (53.5)
Flare immediately prior to tofacitinib initiation, n%				
Yes	376 (60.5)	74 (41.8)	169 (62.1)	133 (76.9)
Symptom severity^b^ prior to tofacitinib initiation, mean (SD)				
Overall symptoms severity				
Missing, n	4	0	4	0
Mean (SD)	3.3 (0.9)	3.2 (1.0)	3.3 (0.8)	3.3 (0.8)
Overall pain				
Missing, n	5	0	4	1
Mean (SD)	3.1 (0.9)	3.1 (1.0)	3.2 (0.8)	3.1 (0.9)
Fatigue/tiredness				
Missing, n	7	2	4	1
Mean (SD)	3.1 (0.9)	3.0 (1.0)	3.2 (0.9)	3.1 (0.9)

Ten patients in the total group excluded from the subgroups as they had either no definite diagnosis date or were initiated on tofacitinib on the day of data collection

Missing data shown only where appropriate

Physician-reported patient data

SD, standard deviation; UC, ulcerative colitis

^a^Two patients had received a Janus Kinase inhibitor at a prior line of treatment (included as part of the biologic treatment line)

^b^Symptom severity prior to initiation: severity score 0, none to 5, extremely severe

^c^Presence of any extra-intestinal manifestations (Musculoskeletal system, Dermatologic and oral systems, Hepatopancreatobiliary system, Ocular system, Metabolic system, Renal system)

At the time of data collection, patients’ mean age was 39.3 (11.3) years, 56.7% were male. The mean (SD) time from diagnosis to tofacitinib initiation was 4.7 (5.5) years; 1.4 (2.2), 5.2 (5.4), and 7.6 (6.2) years in patients who were biologic naïve, had previously received treatment with a single biologic, and had previously received treatment with ≥ 2 biologics, respectively.

The majority of patients (76.2%) had either left-sided (33.0%) or extensive colitis (41.1%). Most frequently, biologic naïve patients (n = 67, 37.4%) had left-sided colitis while patients who had previously received treatment with a single biologic (n = 123, 45.2%) or ≥ 2 biologics (89, 54.9%) had extensive colitis. Approximately half of all patients (n = 292, 45.5%) reported comorbidities; additionally, patients who have received ≥ 2 biologics reported a higher frequency of extra-intestinal manifestations (in any of the following systems: Musculoskeletal system, Dermatologic and oral systems, Hepatopancreatobiliary system, Ocular system, Metabolic system, Renal system).

At tofacitinib initiation, patients most frequently presented with abdominal pain/cramps (n = 486, 76.7%), bloody diarrhea (n = 485, 76.5%), bowel movement urgency (n = 454, 71.6%), and rectal bleeding (n = 335, 52.8%). Frequency of these symptoms increased with number of previously received biologics. Anemia was reported in 162 (25.6%) patients. At initiation, most patients also presented with flare in their UC symptoms, ranging from 40–77% across the treatment groupings. Overall, symptom severity was considered by physicians to be moderate (score 3.1; scale 0, none–5, extremely severe).

### Biologic therapy treatment history and switch to tofacitinib

**Fig. 2:**
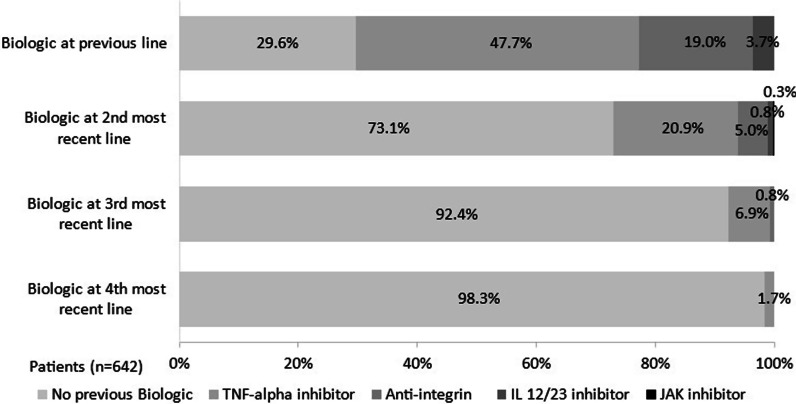
Lines of treatment prior to tofacitinib in patients with moderate-to-severe UC. Legend: Physician-reported patient data (N = 642 at each treatment line). IL, interleukin; JAK, Janus kinase; UC, ulcerative colitis

Of 642 patients, almost 50% of patients on tofacitinib had received a TNFi as their previous biologic therapy; 19.0% and 3.7% of patients had received an anti-integrin and an IL 12/23 antagonist respectively (Fig. 2).

Of 642 patients, physicians reported that their most frequent reasons for choosing tofacitinib for their patients were overall efficacy (71.3% of patients), mode of administration (61.4%), to induce remission (59.2%), and for symptom relief (54.7%). The remaining ‘top ten’ reasons were to maintain remission (47.4%), rapid onset of action (47.2%), reduce the need of steroids (43.1%), treat a flare (42.1%), long-term efficacy (40.5%), and pain relief (39.6%) (Additional file 1, Additional file 2).

### Tofacitinib dose, dose frequency, and dose changes

**Fig. 3:**
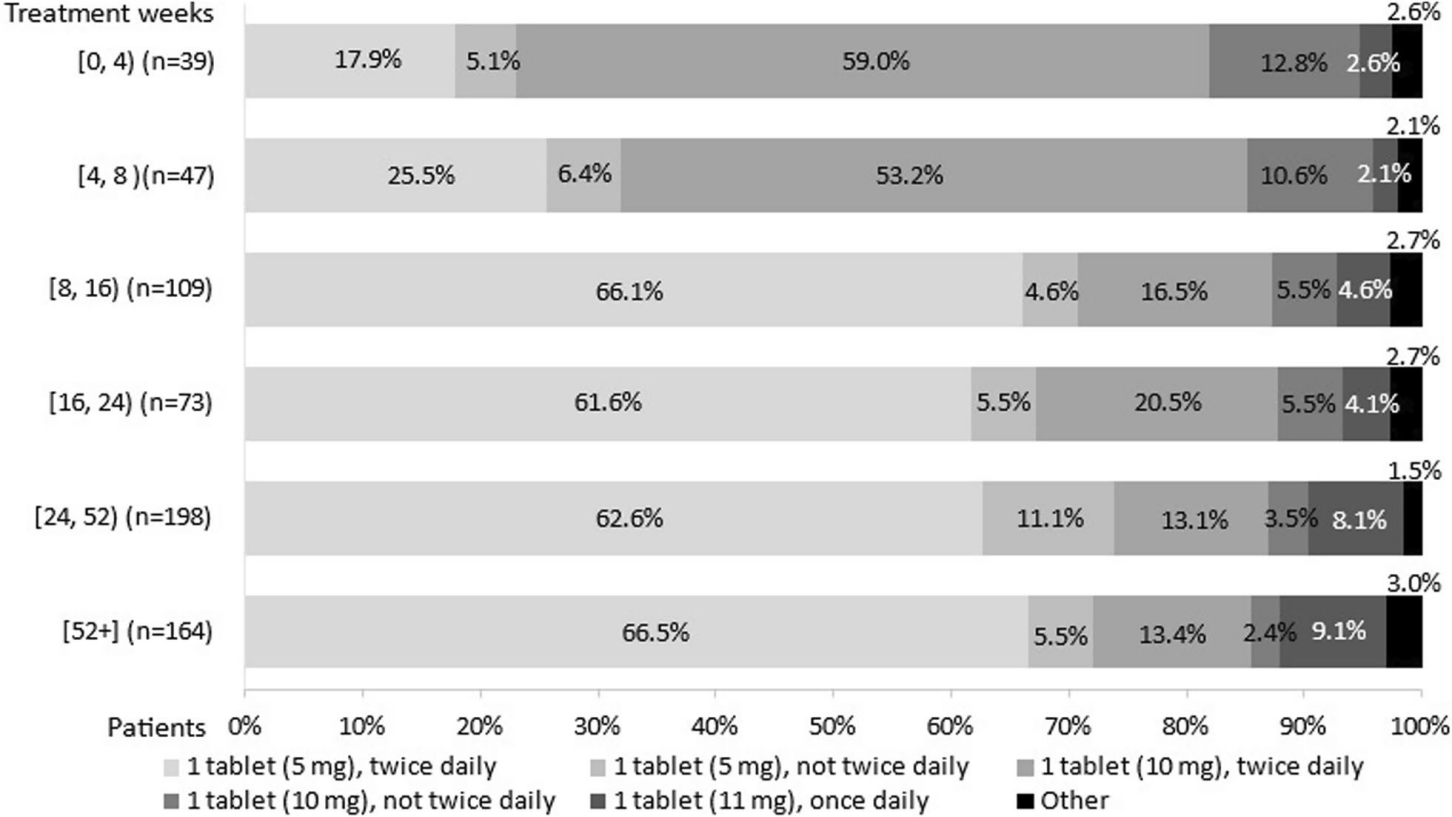
Tofacitinib dose and dose frequency during treatment week in patients with moderate-to-severe UC. Legend: Tofacitinib Induction: Tofacitinib 10 mg twice daily for at least 8 weeks; then 5 or 10 mg twice daily. Discontinue after 16 weeks of 10 mg twice daily, if adequate therapeutic benefit is not achieved. Use the lowest effective dose to maintain response. Maintenance: 5 mg twice daily. Tofacitinib XR: Induction: Tofacitinib XR 22 mg once daily. Maintenance: 11 mg once daily. Other, n = 15 (2.5%): 2 tablets (5 mg), twice daily (n = 8, 1.3); 1 tablet (unspecified), twice daily (n = 1, 0.2%); 1 tablets (11 mg), not once daily (n = 5, 0.8%); 2 tablets (11 mg), once daily (n = 1, 0.2%). Physician-reported patient data. Chi square test: *p* < 0.0001 (The null hypothesis states that the percentage of patients within each dose category is the same for each of the treatment duration groups). UC, ulcerative colitis

Over the treatment period, 630 patients had dosing data (Fig. 3). Ninety-eight patients were in the induction phase at time of data collection, and 544 were in the maintenance phase. Within the induction population the tofacitinib dose and/or frequency was unchanged for 88.8% of patients, escalated for 3.1%, and de-escalated for 8.2% of patients. In the maintenance population, the tofacitinib dose and/or frequency for 19.5%, 7.2% and 73.3% of patients was de-escalated, escalated, and unchanged, respectively.

During the first 8 weeks of treatment, over half of patients were prescribed one 10 mg tablet twice daily, and one-quarter of patients were prescribed a lower dose of one 5 mg tablet twice daily. From week 8 up to week 16, 66.1% and 16.5% of patients were prescribed one 5 mg tablet twice daily and one 10 mg tablet twice daily, respectively. After the first 8 weeks of treatment, > 60% of patients were prescribed one 5 mg tablet twice daily, while between 13.1–20.5% were prescribed a higher dose of one 10 mg tablet twice daily. Less than 10% of patients receiving one 11 mg tablet once daily over the treatment period.

### Disease activity during tofacitinib treatment

**Fig. 4:**
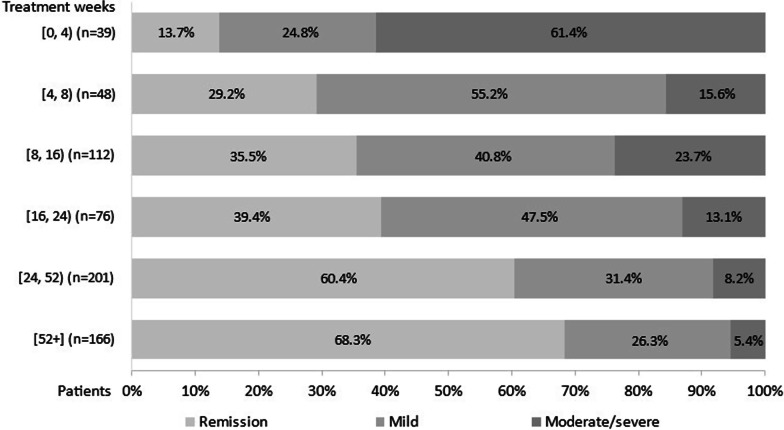
Disease activity (partial Mayo score) in patients with moderate-to-severe UC during tofacitinib treatment. Legend: Disease activity derived from physician-reported data as evaluated by the total partial Mayo disease activity score. Chi square test: *p* < 0.0001. UC, ulcerative colitis

Of the 642 patients, disease severity was moderate/severe in the majority of patients (61.4%) at the initial *[0, 4)* weeks of tofacitinib as determined by partial Mayo. The proportions of patients in remission according to partial Mayo score increased from treatment initiation to *[52*+*]* weeks treatment, with 13.7%, 29.2%, 35.5%, 39.4%, 60.4%, and 68.3% at weeks *[0, 4), [4, 8), [8, 16), [16, 24), [24, 52), and [52*+*]*, respectively (Fig. 4). There was a slight increase in the proportion of patients at weeks *[8, 16)* with moderate/severe disease.

### Steroid history, current steroid use, and disease activity

Among the 642 patients, 153 (23.8%) patients used steroids in the treatment line before initiating tofacitinib, 123 (19.2%) patients discontinued steroids at initiation of tofacitinib, and 30 (4.7%) patients continued to use steroids and were using steroids with tofacitinib at time of data collection. A further 29 (4.5%) patients initiated steroids at tofacitinib initiation and continued to day of data collection. In total, a steroid was used in combination with tofacitinib in 59 (9.2%) patients at the day of data collection.

Over the course of the tofacitinib plus steroid treatment regimen, the steroid dose was de-escalated (reduced dose and/or frequency) in 45% of patients or remained the same/fluctuated in 50% of the 59 patients (Additional file 3).

### Symptom improvement following tofacitinib initiation

#### Perspectives of physicians and patients of symptoms

**Fig. 5:**
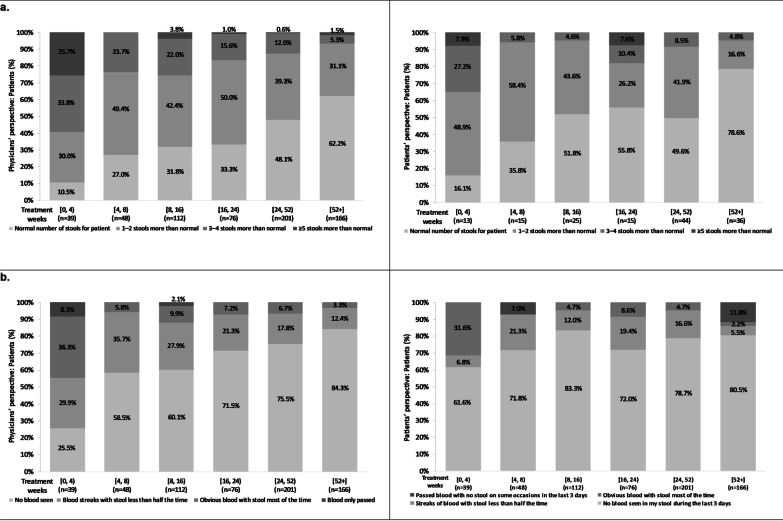
Physician- and patient-reported symptoms in patients with moderate-to-severe UC by time on tofacitinib. **a** Stool frequency; **b** Bloody stool presence. Legend: Least squares mean percent adjusted for age, sex, body mass index, Charlson comorbidity index, and number of previous biologic treatments. Stool frequency: Physicians’ and patients’ perspectives: Wald test: *p* < 0.0001; Bloody stool presence: Physicians’ perspective: Wald test: *p* < 0.0001; Patients’ perspective: *p* value could not be generated due to low patient numbers. UC, ulcerative colitis

Both physicians and patients reported that stool frequency (obtained as a component of the Mayo score) was reduced with time on treatment. Physicians reported an increase from 10.5 to 62.2% of patients with a normal number of stools from weeks *[0, 4)* to *[52*+*]*, and from 16.1 to 78.6% of patients reported a normal number of stools from weeks *[0, 4)* to *[52*+*]* (Fig. 5a). Generally, the proportion of patients with 3–4 or ≥ 5 stools more than normal was over-estimated by physicians according to patient-reported stool frequency. Notably, during the first 4 weeks after treatment initiation, ≥ 5 stools more than normal was reported for 25.7% of patients by physicians vs. 7.9% by patients.

The presence of blood in stool (obtained as a component of the Mayo score) was also reduced with time on treatment (Fig. 5b). Physicians (n = 642) reported a decrease from 74.5 to 15.7% of patients seeing blood (including streaks of blood with stool less than half the time, obvious blood with stool most of the time and blood alone passed when passing stools) from weeks *[0, 4)* to *[52*+*]*; there was a decrease in patients (n = 147) reporting the presence of blood seen in their stool over the past 3 days from weeks *[0, 4)* to *[52*+*]* from 38.4 to 19.5%. Thus, physicians over-estimated blood in stool at treatment start. However, the proportion of patients with blood in stool most of the time or passing blood only was under-estimated by physicians at weeks *[52*+*]* according to patients-reported data.

#### Symptom change in the five most frequently reported symptoms

**Fig. 6:**
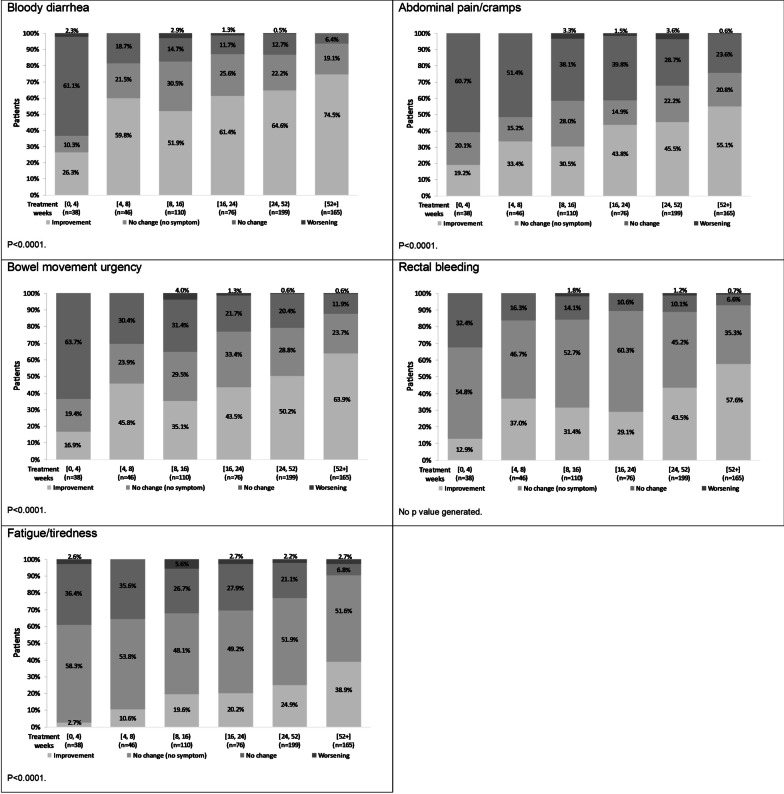
Symptom change in the most frequently reported symptoms among patients with moderate-to-severe UC following tofacitinib initiation. Legend: Physician-reported patient data. No change (no symptom) indicates patients who had no symptoms continued to show no symptoms; No change indicates patients who had symptoms continued to show symptoms. Least squares mean percent adjusted for age, sex, body mass index, Charlson comorbidity index, and number of previous biologic treatments. Wald test: *p* < 0.0001. UC, ulcerative colitis

Symptom change was examined in bloody diarrhea, abdominal pain/cramps, bowel movement urgency, rectal bleeding, and fatigue/tiredness (Fig. 6). Symptom improvement for all of these symptoms was reported in the first weeks of treatment, with the proportion of patients with improvement increasing with length of time on tofacitinib. No change was reported for the majority of patients with no symptoms (i.e., they continued to show no symptoms), particularly rectal bleeding and fatigue/tiredness. Overall, < 5% of patients had worsening of symptoms at any one time period.

#### Change in overall severity of symptoms, pain, and fatigue/tiredness

A substantial reduction in patients’ mean overall severity of symptoms, pain and fatigue from the tofacitinib treatment initiation to the current (data collection) was reported by physicians (Additional file 4). At week *[52*+*]*, the mean reduction from treatment initiation to current in overall severity of symptoms was 2.2 (to a current mean score of 1.1), in pain was 2.2 (to a current mean score of 0.9), and in fatigue was 2.1 (to a current mean score of 1.0), respectively.

### Patient-reported outcomes

Improvement was shown in all patient-reported outcomes from week 4– < 8 that continued with increasing time on tofacitinib (Additional file 5; all patient-reported outcomes, *p* < 0.0001). Comparing patients at weeks *[0, 4)* and *[52*+*]*, the total increase in EQ-5D-5L index total score was 0.29 points and in SIBDQ total score was 20.5 points, and the reductions in WPAI percent absenteeism was 34.4%, presenteeism was 26.8%, overall work impairment was 40.9% and activity impairment was 28.3%. These changes across weeks *[0, 4)* to *[52*+*]* reached the assessments’ MCID.

Minimal clinically important differences were found between patients at weeks *[0, 4)* and *[4, 8)* in the reductions in EQ-5D-5L index total score (0.09 points), and WPAI percent absenteeism (17.5%), presenteeism (11.7%), and overall work impairment (24.6%); the reduction in SIBDQ total score (8.3 points) nearly reached the threshold of 9 points for clinical difference. Clinically important differences were found between patients at weeks *[0, 4)* and *[8, 16)* in the reductions in SIBDQ total score (9.7 points) and the WPAI percent activity impairment (14.3%).

### Physician and patient satisfaction with treatment

**Fig. 7:**
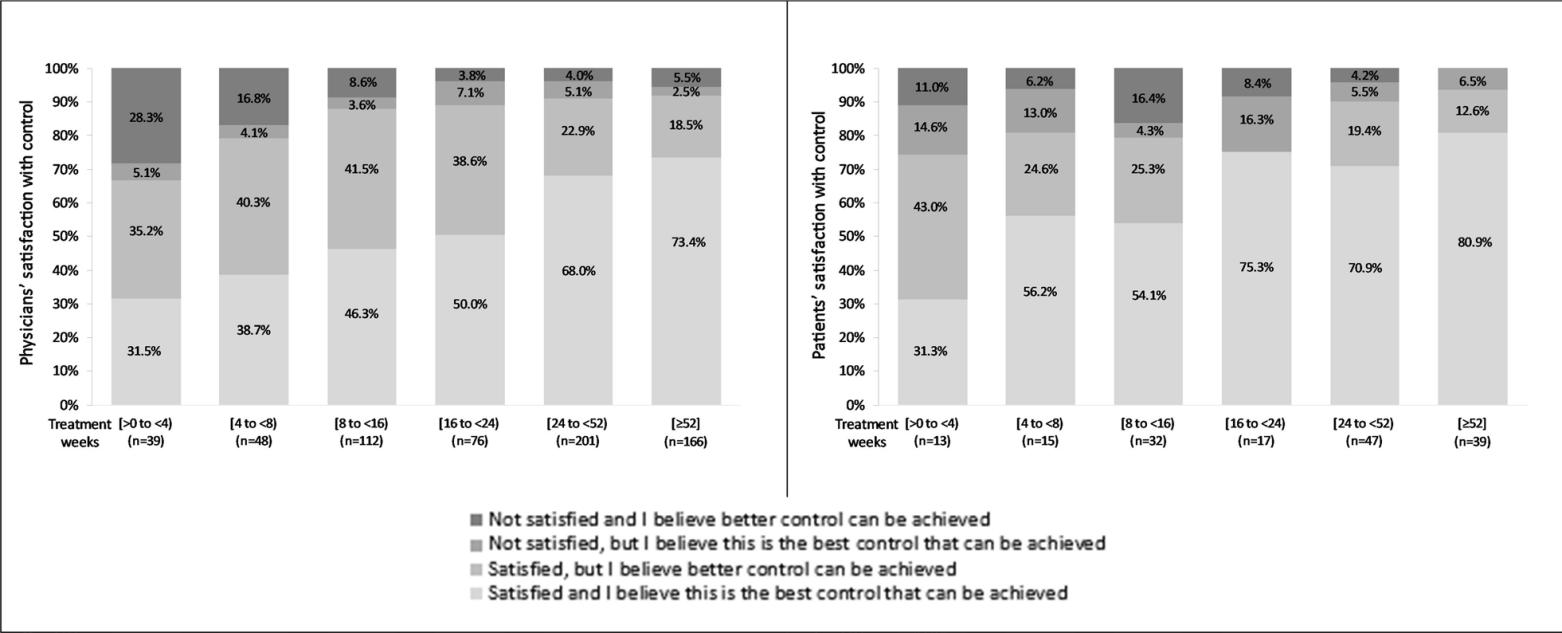
Satisfaction with tofacitinibib treatment among physicians and their patient with moderate-to-severe UC. Legend: Least squares mean percent adjusted for age, sex, body mass index, Charlson comorbidity index, and number of previous biologic treatments. Physicians’ and patients’ satisfaction, Wald test: *p* < 0.0001. UC, ulcerative colitis

The proportions at physicians and patients who were satisfied with tofacitinib treatment was highest among those who had *[52*+*]* weeks of treatments compared with those at [0, 4] weeks (Fig. 7). The majority of patients and physicians were satisfied with tofacitinib treatment. At weeks *[0, 4)*, > 66.7% of physicians and > 74.3% of patients were satisfied with tofacitinib, increasing to 91.9% and 93.5%, respectively, at weeks *[52*+*]*.

## Discussion

This analysis of the Adelphi UC DSP describing clinical characteristics, variations in treatment patterns, and treatment outcomes in patients with UC receiving tofacitinib found that overall, the majority of physicians followed treatment recommendations at induction and through maintenance; that disease activity, symptoms frequency and severity is reduced; and that high rates of remission were achieved. QoL is improved and treatment satisfaction were achieved during treatment over the ≥ 52-weeks assessment period in patients with moderate-to-severe UC treated with tofacitinib in a real-world setting. Our real-life data support the clinical efficacy of tofacitinib as observed in RCTs [16].

Biologics were used early in treatment of moderate-to-severe UC, with the use of TNFi therapy across subsequent lines decreasing as newer therapies became available. A previous analysis of the Adelphi Inflammatory Bowel Disease DSP in 2017 characterised the increase in use of biologic therapy in patients with UC [34]. The cycling across multiple biologics seen from this study may be attributed to patients having both primary non-response and secondary non-response [12]. This was supported by our findings which found that the reason physicians most frequently chose tofacitinib was for its efficacy and for rapid symptom relief, suggesting primary or secondary non-response, particularly to TNFi therapy. Although this study did not capture reasons for previous biologic failure, these reasons may be a factor for how patients will respond to future treatments. In a network meta-analysis of RCTs of patients with inflammatory bowel disease reported that patients who discontinued their first-line TNFi therapy due to primary non-response were less likely to respond to second-line biologics compared to those who had secondary loss of response or intolerance to TNFi [11].

Prescribing dosage of tofacitinib typically followed treatment recommendations, with few dose changes reported, particularly during induction. While the dose remained unchanged for the majority of patients during maintenance, changes in dose typically involved de-escalation to a lower dose or lower dose frequency. It is recommended that the lowest effective dose is typically used, however it is possible that dose changes were due to intolerance, loss of response or non-response, leading to dose de-escalation and dose escalation, respectively. An observational study also noted dose reductions in patients continuing tofacitinib after week 24 [35]. Analysis of data from the randomised, double-blind RIVETING trial of tofacitinib dose reduction to 5 mg twice daily versus remaining on 10 mg twice daily, found that most patients in stable remission on 10 mg twice daily maintenance therapy maintained remission following dose de-escalation [36]. Additionally, in contrast to biologic therapies that are liable to loss of exposure due to substantial disease activity or immunogenicity [37], the pharmacokinetic results in the OCTAVE Induction 1 and 2 and Sustain trials found no indication of a decrease in plasma tofacitinib concentrations during the treatment course at any dose in individual patients [16].

Our analysis demonstrated that the number of patients in remission was positively correlated with length of time on tofacitinib treatment, with the proportion of patients increasing by 54% from initiation to weeks *[52*+*]*, possibly suggesting that these patients may be those who continued with tofacitinib treatment and were early responders. Specifically, at weeks *[4, 8)* less than one-third (29.2%) of patients were in remission; the proportion was more than two-thirds (68.3%) of patients at weeks > 52. A meta-analysis of real-world studies evaluating the effectiveness of tofacitinib for moderate-to-severely active UC, remission was achieved in 34.7% (95% confidence interval [CI], 24.4–45.1%), 47.0% (95% CI 40.3–53.6%), and 38.3% (95% CI 29.2–47.5%) of patients at week 8, weeks 12–16, and month 6, respectively [38]. These real-world data also support findings of the OCTAVE Induction 1 and 2 trials, where 18.5% and 16.6% of patients with UC treated with tofacitinib (versus 8.2% and 3.6% receiving placebo), respectively, achieved clinical remission by week 8 [16]. During the maintenance OCTAVE Sustain trial, remission occurred in 34.3% of patients in the 5 mg tofacitinib group and 40.6% in the 10 mg tofacitinib group (versus 11.1% placebo) group at 52 weeks [16].

The gastrointestinal symptoms associated with UC significantly impact on patients’ QoL [6]. There are currently limited real-world studies that have assessed improvement in individual symptoms and QoL with tofacitinib as in our cross-sectional study [39]. Our analysis demonstrated that tofacitinib improves symptoms, symptom severity, and QoL in patients with moderate-to-severe UC, with improvements beginning in the first month of treatment and increasing with length of time on tofacitinib therapy. Thus, we found a reduction in the proportion of patients with more stools than normal and/or with bloody stools, with the vast majority reporting normal non-bloody stools at/after a year of treatment. Similarly, other gastrointestinal symptoms (bloody diarrhea, abdominal pain/cramps, bowel movement urgency, rectal bleeding) and fatigue/tiredness showed improvement with length of time on tofacitinib, and overall severity of symptoms, pain and fatigue/tiredness were substantially reduced from tofacitinib initiation. QoL showed clinically meaningful improvements as early as the second or third month of treatment. Specifically, the improvement in work productivity, amounting to over 40% overall is an important finding for patients with UC of working age. Over 90% of people with IBD who have symptoms during their working life require workplace accommodations to enable them to work, yet many found them difficult to arrange or did not ask for them [40].

Our findings generally concur with RCTs during induction and maintenance, as we found improvement in QoL in both these treatment periods, with the greatest improvements observed during induction. In the OCTAVE induction studies, there were statistically significant and clinically meaningful improvements in HRQoL of patients with moderate-to-severe UC with tofacitinib (versus placebo) as early as Week 4, with sustained improvements in the maintenance study [18]. Similarly, a systematic review with network meta-analysis of RCTs comparing the impact of therapies on HRQL found that induction therapy with tofacitinib improves HRQoL of patients with moderate-to-severe UC, with the improvement maintained during maintenance therapy [41].

The majority of physicians and patients were satisfied with tofacitinib, starting from treatment initiation, and rising to over 90% of individuals from 6 months onwards; similar patterns were seen for physicians and patients over time in terms of satisfaction. A survey of 194 patients with UC receiving tofacitinib 10 mg twice daily for 8 weeks reported that approximately 60% of patients were each at least satisfied with tofacitinib, preferred tofacitinib to their prior treatment, and would use tofacitinib again [42]. In agreement, we found 79% of patients were satisfied with tofacitinib at *[4, 8)* weeks. Supporting our findings, bowel function has been shown to be important for patient treatment satisfaction with tofacitinib [43].

By design, DSPs are non-interventional and capture data from a large representative patient sample in a real-world setting. Nevertheless, several limitations should be considered in the evaluation of our findings. Participating patients may not reflect the general UC population as they may be the more frequently consulting patients who may have had more active disease. However, identification of patients with UC is based on the judgement of the physician and is representative of physician’s real-world clinical practice. Consecutively presenting patients were included so that a patient consultation is at random, although the patient samples were not truly random as the methodology dictates inclusion of the next ‘n’ number of consecutive consulting patients, and there are no data verification procedures for choosing the consecutive series of patients. Physician inclusion was likely influenced by their willingness to participate. Physician- and patient-reported data may be subject to recall bias; this may have affected responses particularly of time-stamped data such as steroid use, which therefore may be under-estimated. Not all patients completed a PSC, so there is a disconnect between available physician reported and patient reported data. The comparisons are between the independent groups of patients across time. The cross-sectional nature of the DSP means that the data were captured at a single point in time and do not follow individuals up over time; thus, there can be no temporal relationship between exposure and outcome. Note the MCIDs used here to contextualise the mean differences in some patient reported outcomes between patient groups are intended to give context to changes within individual patients. Finally, we cannot rule out any impact on our findings of COVID-19, since data were collected during the pandemic.

## Conclusions

In conclusion, this cross-sectional real-world analysis demonstrates that patients with moderate-to-severe UC treated with tofacitinib show considerable improvement in symptoms and HRQoL from tofacitinib initiation though maintenance to one year and beyond, with approximately 60% of patients attaining remission at weeks 24–52. Physicians and patients report satisfaction with UC control at recommended doses early in treatment despite patients’ previous treatment lines. Our findings highlight the importance of real-world studies to demonstrate outcomes of the every-day patient population outside the stringent eligibility criteria of clinical trials.

## Supplementary Information

**Additional file 1**. Most frequent reasons for choosing biologic therapy at previous treatment lines for patients with moderate-to-severe UC. Physician-reported patient data. Two patients had received a Janus Kinase inhibitor at a prior line of treatment (included as part of the biologic treatment line). UC, ulcerative colitis.

**Additional file 2**. Reasons for choice of tofacitinib in patients with moderate-to-severe UC by length of time on tofacitinib. UC, ulcerative colitis.

**Additional file 3**. Changes in steroid dose/frequency during the course of the treatment regimen in patients with moderate-to-severe UC on tofacitinib. UC, ulcerative colitis.

**Additional file 4**. Change in severity of symptoms, pain and fatigue among patients with moderate-to-severe UC from tofacitinib initiation. **a** Overall symptom severity; **b** Overall pain severity; **c** Overall fatigue/tiredness severity. Physician-reported patient data. One observation per patient; the groups are mutually exclusive. Patients’ clinical severity was rated at the start of tofacitinib treatment (i.e., status when on prior treatment) and currently (at data collection) on a scale ranging from 0 (none) to 5 (extremely severe). UC, ulcerative colitis.

**Additional file 5**. Patient-reported outcomes in patients with moderate-to-severe UC. **a** EQ-5D-5L Index total score (US tariff); **b** SIBDQ total score; **c** WPAI component scores. Base sizes for the four WPAI components varied. One observation per patient. Linear regression with categorised time and additional covariates included. The EQ-5D index total score ranges from < 0.00 to 1.00, where higher scores indicate better HRQL; a 0.074-point change in the EQ-5D scale is considered a MCID. The SIBDQ total score ranges from 10 indicating worst health to 70 indicating best health; a 9-point change in the SIBDQ is considered the MCID. The WPAI component ranges from 0%, no impairment to 100%, total loss of work productivity or activity; a change of 6.5%, 6.1%, 7.3%, and 8.5% are considered to be MCIDs for absenteeism, presenteeism, overall work impairment, and total activity impairment, respectively. EQ-5D-5L, EuroQol-5 Dimension-5 Level; MCID, minimal clinically important difference; SE, standard error; SIBDQ, short version of the Inflammatory Bowel Disease Questionnaire; WPAI, Work Productivity and Activity Impairment. UC, ulcerative colitis.

## Data Availability

All data, i.e. methodology, materials, data and data analysis, that support the findings of this survey are the intellectual property of Adelphi Real World. All requests for access should be addressed directly to Benjamin Hoskin (ben.hoskin@adelphigroup.com).

## Abbreviations

DSP: Disease Specific Programme
EQ-5D-5L: EuroQol-5 Dimension-5 Level
HRQoL: Health-related quality of life
IL: Interleukin
JAK: Janus kinase
MCID: Minimal clinically important difference
QoL: Quality of life
RCT: Randomised controlled trials
TNFi: Tumour necrosis factor inhibitors
SIBDQ: Short version of the Inflammatory Bowel Disease Questionnaire
UC: Ulcerative colitis
WPAI-SHP: Work Productivity and Activity Impairment questionnaire-specific health problem
