# Supplementary material for: Characteristics, clinical outcomes and patient-reported outcomes of patients with ulcerative colitis receiving tofacitinib: a real-world survey in the United States and five European countries
Source: BMC Gastroenterol. 2023 Jan 19;23:17. doi: 10.1186/s12876-023-02640-7 (PMC9849840; doi:10.1186/s12876-023-02640-7)
Supplement: Supplementary file 1 — Additional file 1. Most frequent reasons for choosing biologic therapy at previous treatment lines for patients with moderate-to-severe UC. Physician-reported patient data. Two patients had received a Janus Kinase inhibitor at a prior line of treatment (included as part of the biologic treatment line). UC, ulcerative colitis. [file 12876_2023_2640_MOESM1_ESM.docx]

**Additional file 1.** DOC. Most frequent reasons for choosing biologic therapy at previous treatment lines for patients with moderate-to-severe UC

| **Reason, n (%)** | **Total (N=642)** | **First biologic  (N=190)** | **Second biologic (N=279)** | **Third biologic and beyond (N=173)** |
| --- | --- | --- | --- | --- |
| Overall efficacy | 458 (71.3) | 132 (69.5) | 203 (72.8) | 123 (71.1) |
| Mode of administration | 394 (61.4) | 93 (48.9) | 184 (65.9) | 117 (67.6) |
| Induce remission | 380 (59.2) | 95 (50.0) | 177 (63.4) | 108 (62.4) |
| Symptom relief | 351 (54.7) | 101 (53.2) | 165 (59.1) | 85 (49.1) |
| Maintain remission | 304 (47.4) | 66 (34.7) | 149 (53.4) | 89 (51.4) |
| Rapid onset of action | 303 (47.2) | 83 (43.7) | 130 (46.6) | 90 (52.0) |
| Reduces the need of steroids | 277 (43.1) | 59 (31.1) | 127 (45.5) | 91 (52.6) |
| Treat a flare | 270 (42.1) | 61 (32.1) | 124 (44.4) | 85 (49.1) |
| Long-term efficacy | 260 (40.5) | 71 (37.4) | 126 (45.2) | 63 (36.4) |
| Pain relief | 254 (39.6) | 81 (42.6) | 120 (43.0) | 53 (30.6) |

Physician-reported patient data.

Two patients had received a Janus Kinase inhibitor at a prior line of treatment (included as part of the biologic treatment line).

UC, ulcerative colitis.
