# Supplementary material for: Characteristics, clinical outcomes and patient-reported outcomes of patients with ulcerative colitis receiving tofacitinib: a real-world survey in the United States and five European countries
Source: BMC Gastroenterol. 2023 Jan 19;23:17. doi: 10.1186/s12876-023-02640-7 (PMC9849840; doi:10.1186/s12876-023-02640-7)
Supplement: Supplementary file 2 — Additional file 2. Reasons for choice of tofacitinib in patients with moderate-to-severe UC by length of time on tofacitinib. UC, ulcerative colitis. [file 12876_2023_2640_MOESM2_ESM.docx]

**Additional file 2.** DOC**.** Reasons for choice of tofacitinib in patients with moderate-to-severe UC by length of time on tofacitinib


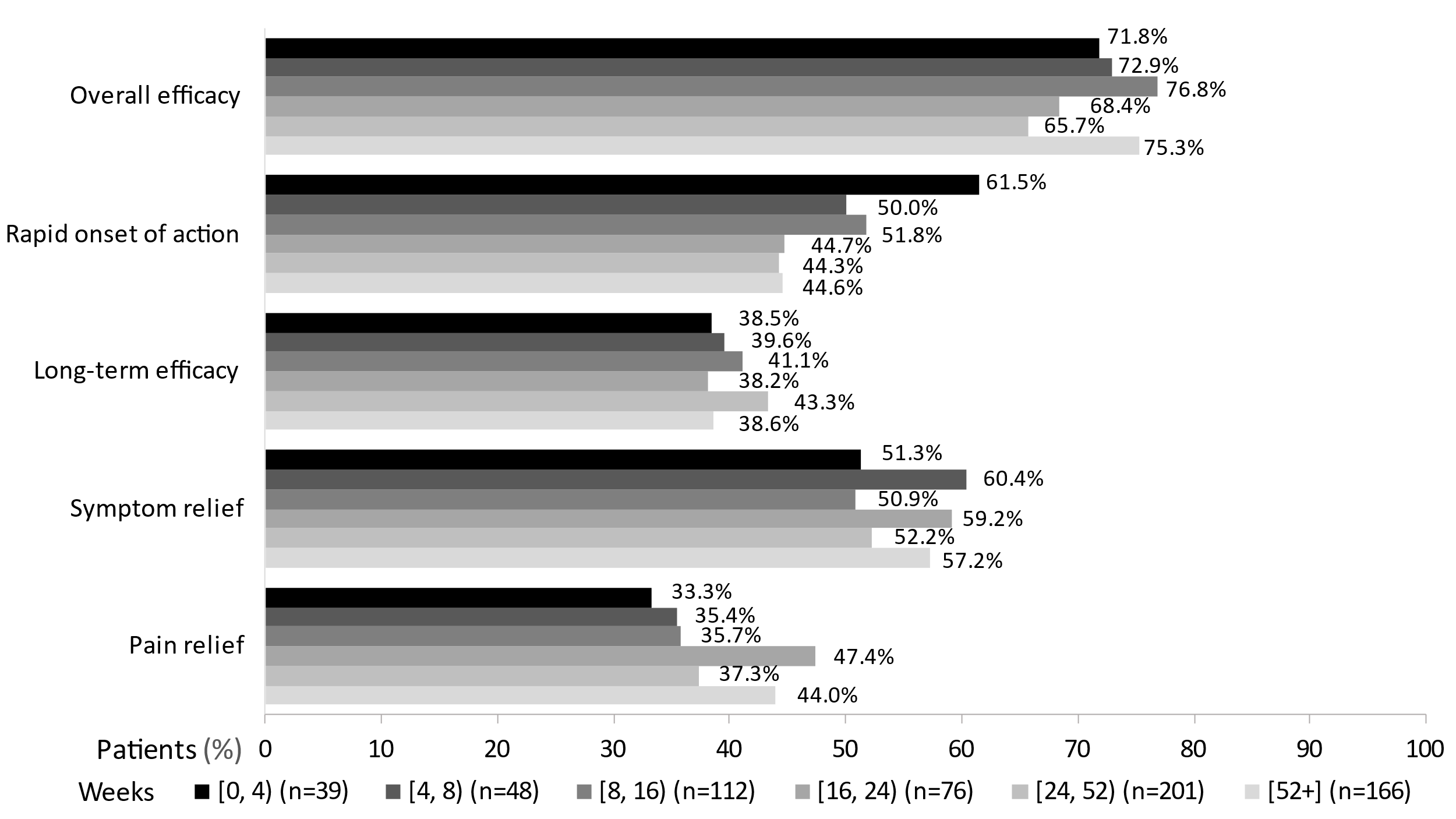


UC, ulcerative colitis.
