# Supplementary material for: Characteristics, clinical outcomes and patient-reported outcomes of patients with ulcerative colitis receiving tofacitinib: a real-world survey in the United States and five European countries
Source: BMC Gastroenterol. 2023 Jan 19;23:17. doi: 10.1186/s12876-023-02640-7 (PMC9849840; doi:10.1186/s12876-023-02640-7)
Supplement: Supplementary file 3 — Additional file 3. Changes in steroid dose/frequency during the course of the treatment regimen in patients with moderate-to-severe UC on tofacitinib. UC, ulcerative colitis. [file 12876_2023_2640_MOESM3_ESM.docx]

**Additional file 3.** DOC. Changes in steroid dose/frequency during the course of the treatment regimen in patients with moderate-to-severe UC on tofacitinib.


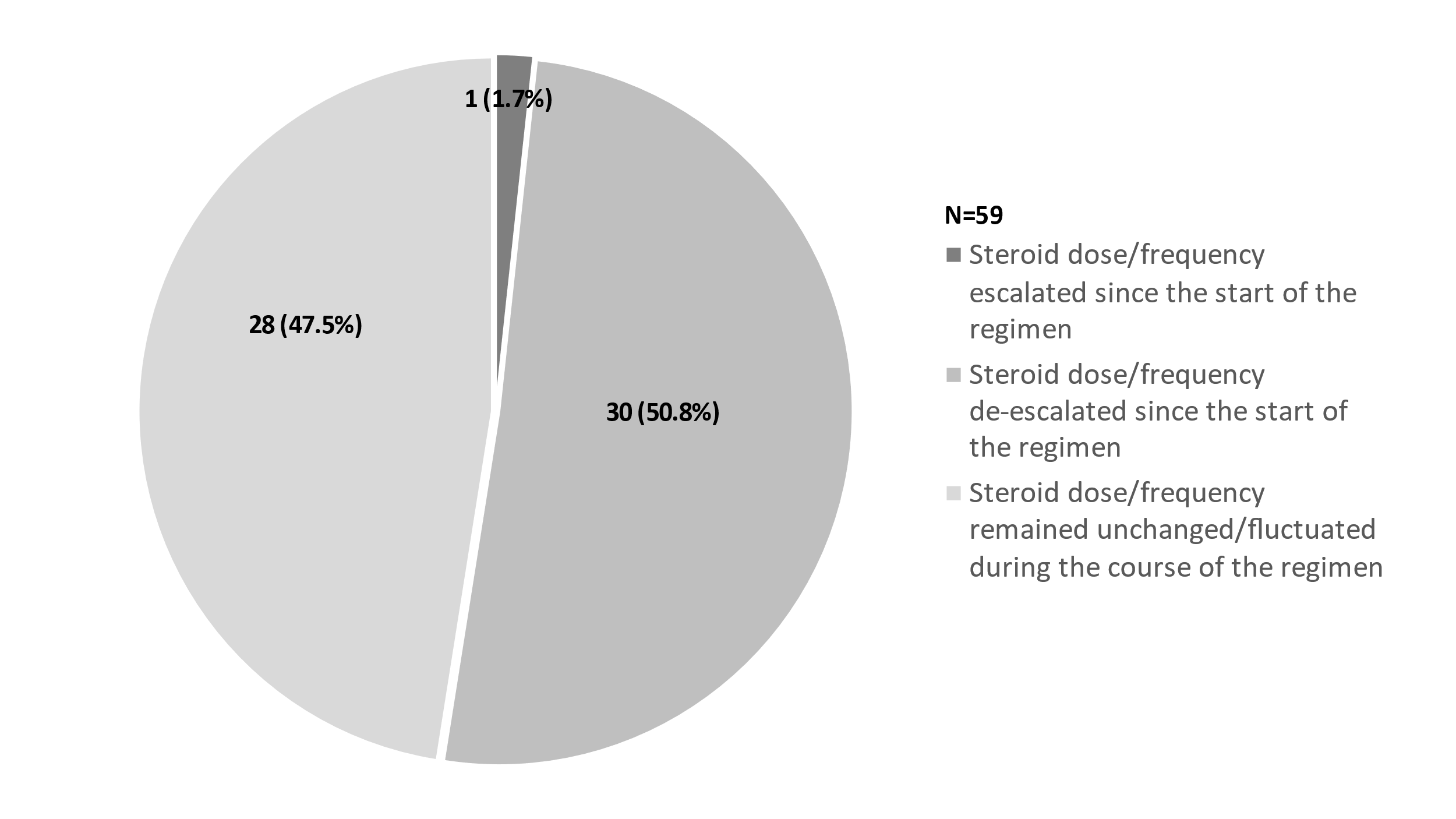


UC, ulcerative colitis.
