# Supplementary material for: Characteristics, clinical outcomes and patient-reported outcomes of patients with ulcerative colitis receiving tofacitinib: a real-world survey in the United States and five European countries
Source: BMC Gastroenterol. 2023 Jan 19;23:17. doi: 10.1186/s12876-023-02640-7 (PMC9849840; doi:10.1186/s12876-023-02640-7)
Supplement: Supplementary file 4 — Additional file 4. Change in severity of symptoms, pain and fatigue among patients with moderate-to-severe UC from tofacitinib initiation. a Overall symptom severity; b Overall pain severity; c Overall fatigue/tiredness severity. Physician-reported patient data. One observation per patient; the groups are mutually exclusive. Patients’ clinical severity was rated at the start of tofacitinib treatment (i.e., status when on prior treatment) and currently (at data collection) on a scale ranging from 0 (none) to 5 (extremely severe). UC, ulcerative colitis. [file 12876_2023_2640_MOESM4_ESM.docx]

**Additional file 4.** DOC. Change in severity of symptoms, pain and fatigue among patients with moderate-to-severe UC from tofacitinib initiation. **a** Overall symptom severity; **b** Overall pain severity; **c** Overall fatigue/tiredness severity

**a.**


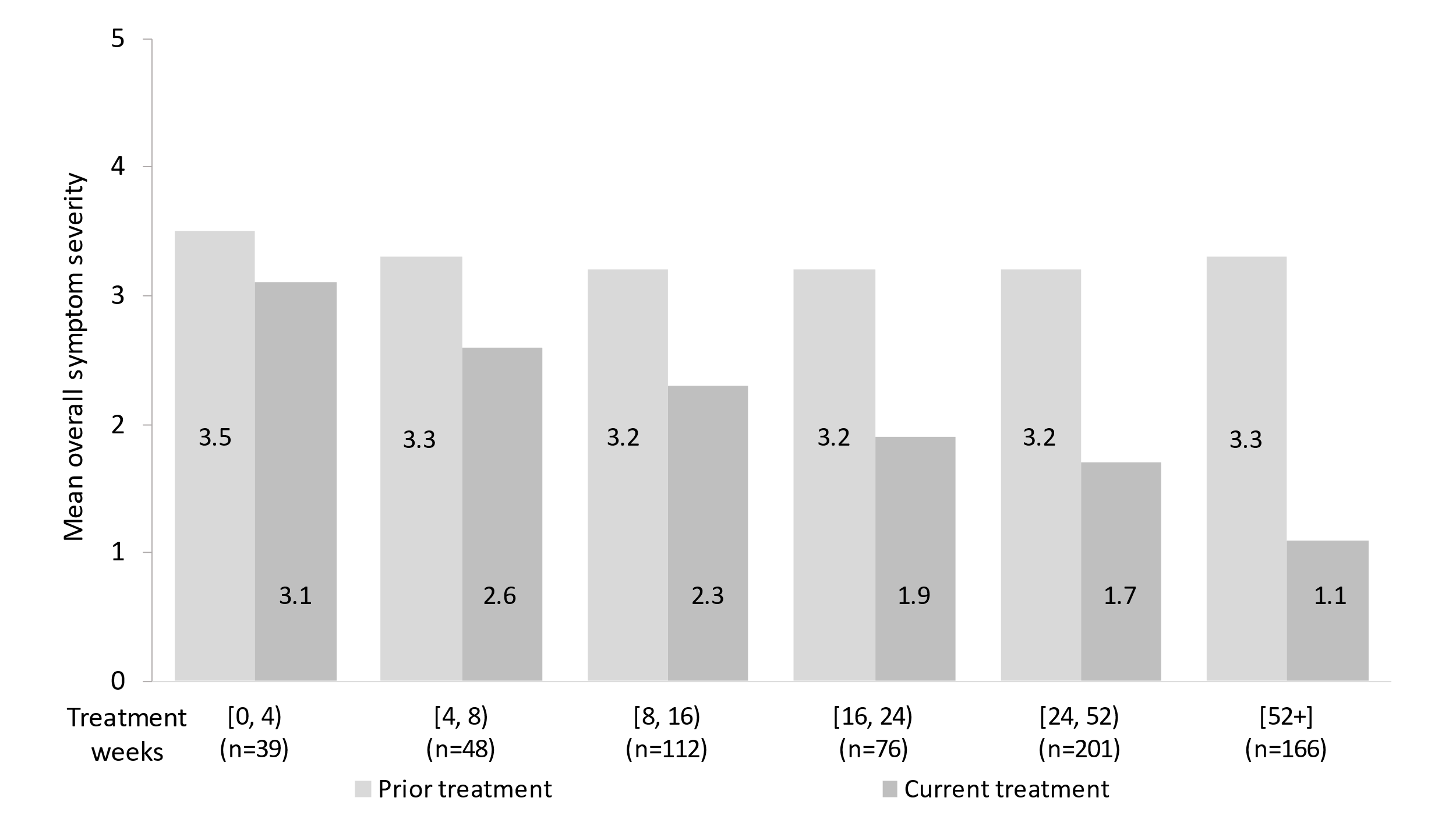


.

**b.**
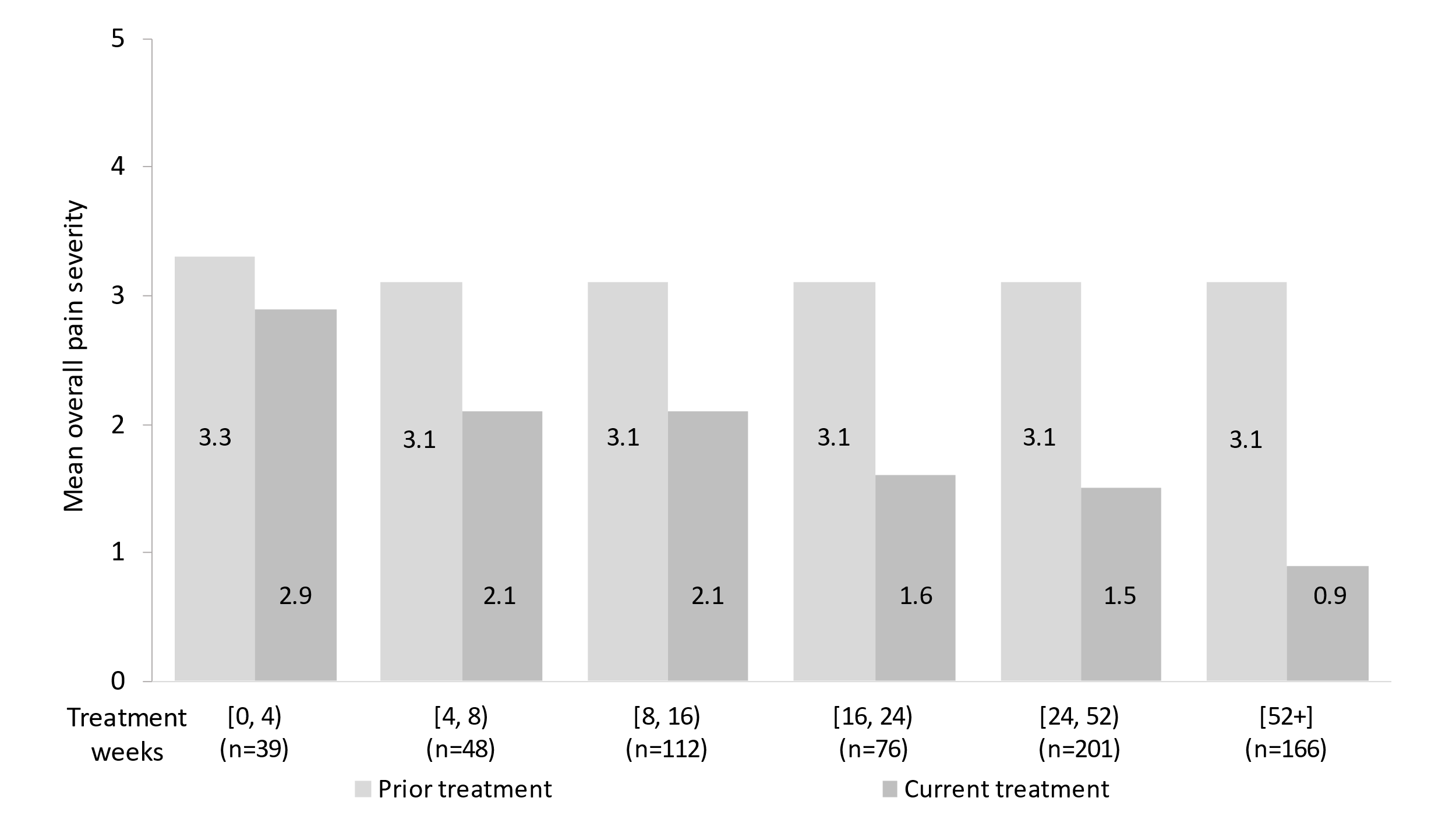


.

**c.**


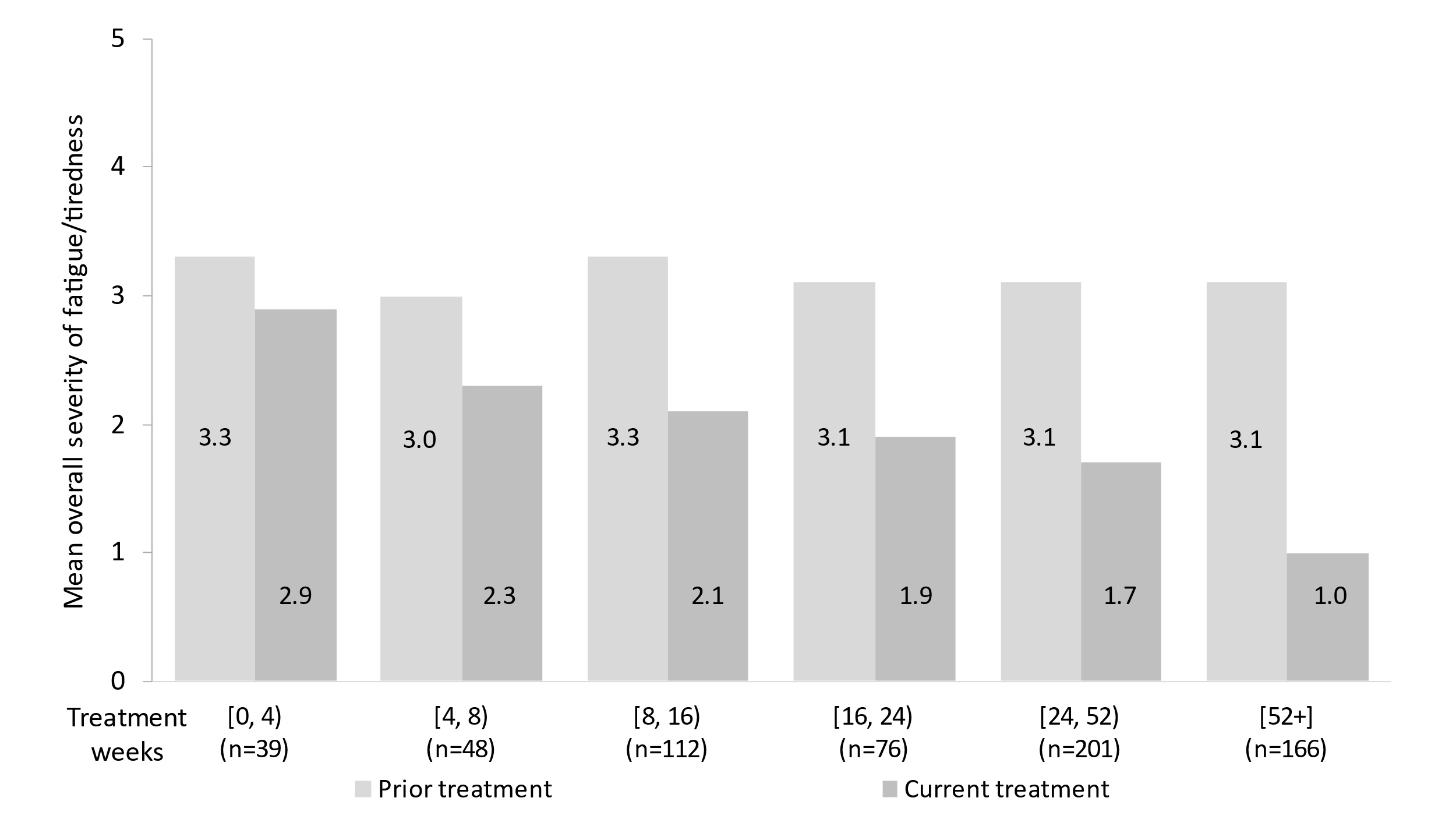


Physician-reported patient data. One observation per patient; the groups are mutually exclusive.

Patients’ clinical severity was rated at the start of tofacitinib treatment (i.e., status when on prior treatment) and currently (at data collection) on a scale ranging from 0 (none) to 5 (extremely severe).

UC, ulcerative colitis.
