# Supplementary material for: Characteristics, clinical outcomes and patient-reported outcomes of patients with ulcerative colitis receiving tofacitinib: a real-world survey in the United States and five European countries
Source: BMC Gastroenterol. 2023 Jan 19;23:17. doi: 10.1186/s12876-023-02640-7 (PMC9849840; doi:10.1186/s12876-023-02640-7)
Supplement: Supplementary file 5 — Additional file 5. Patient-reported outcomes in patients with moderate-to-severe UC. a EQ-5D-5L Index total score (US tariff); b SIBDQ total score; c WPAI component scores. Base sizes for the four WPAI components varied. One observation per patient. Linear regression with categorised time and additional covariates included. The EQ-5D index total score ranges from < 0.00 to 1.00, where higher scores indicate better HRQL; a 0.074-point change in the EQ-5D scale is considered a MCID. The SIBDQ total score ranges from 10 indicating worst health to 70 indicating best health; a 9-point change in the SIBDQ is considered the MCID. The WPAI component ranges from 0%, no impairment to 100%, total loss of work productivity or activity; a change of 6.5%, 6.1%, 7.3%, and 8.5% are considered to be MCIDs for absenteeism, presenteeism, overall work impairment, and total activity impairment, respectively. EQ-5D-5L, EuroQol-5 Dimension-5 Level; MCID, minimal clinically important difference; SE, standard error; SIBDQ, short version of the Inflammatory Bowel Disease Questionnaire; WPAI, Work Productivity and Activity Impairment. UC, ulcerative colitis. [file 12876_2023_2640_MOESM5_ESM.docx]

**Additional file 5.** DOC. Patient-reported outcomes in patients with moderate-to-severe UC. **a** EQ-5D-5L Index total score (US tariff); **b** SIBDQ total score; **c** WPAI component scores

**a.**


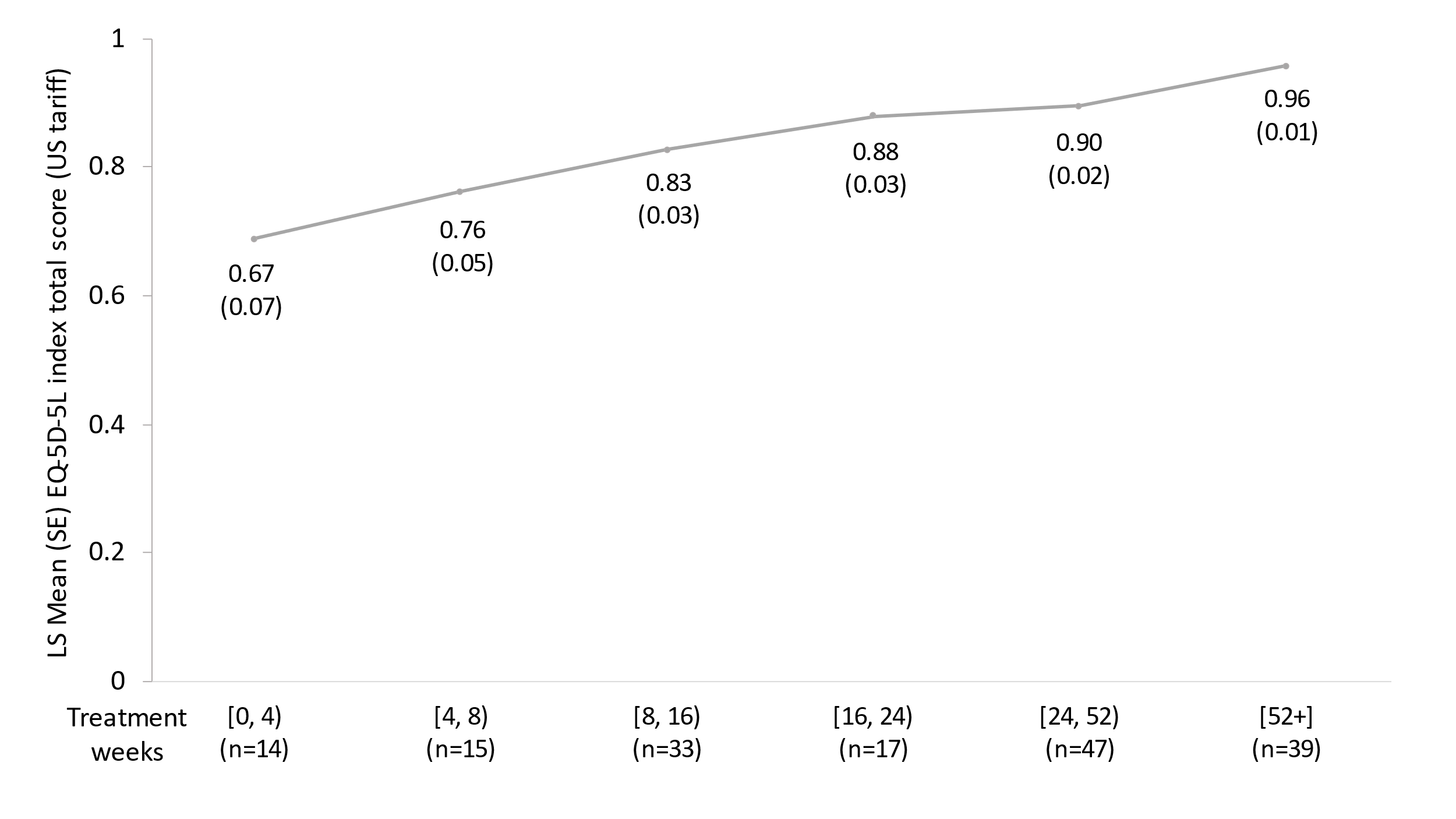


.

**b.**


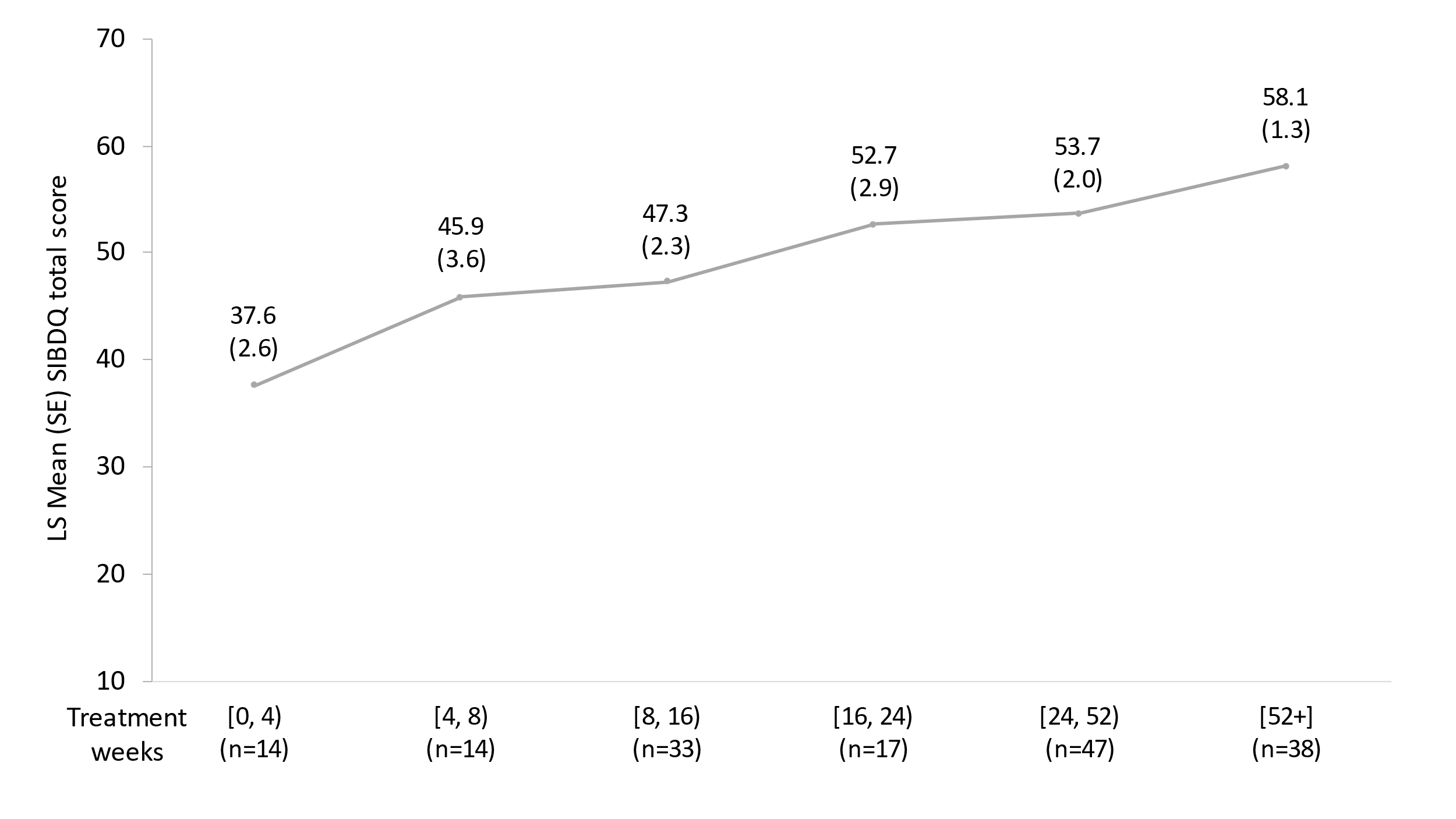


.

**c.**


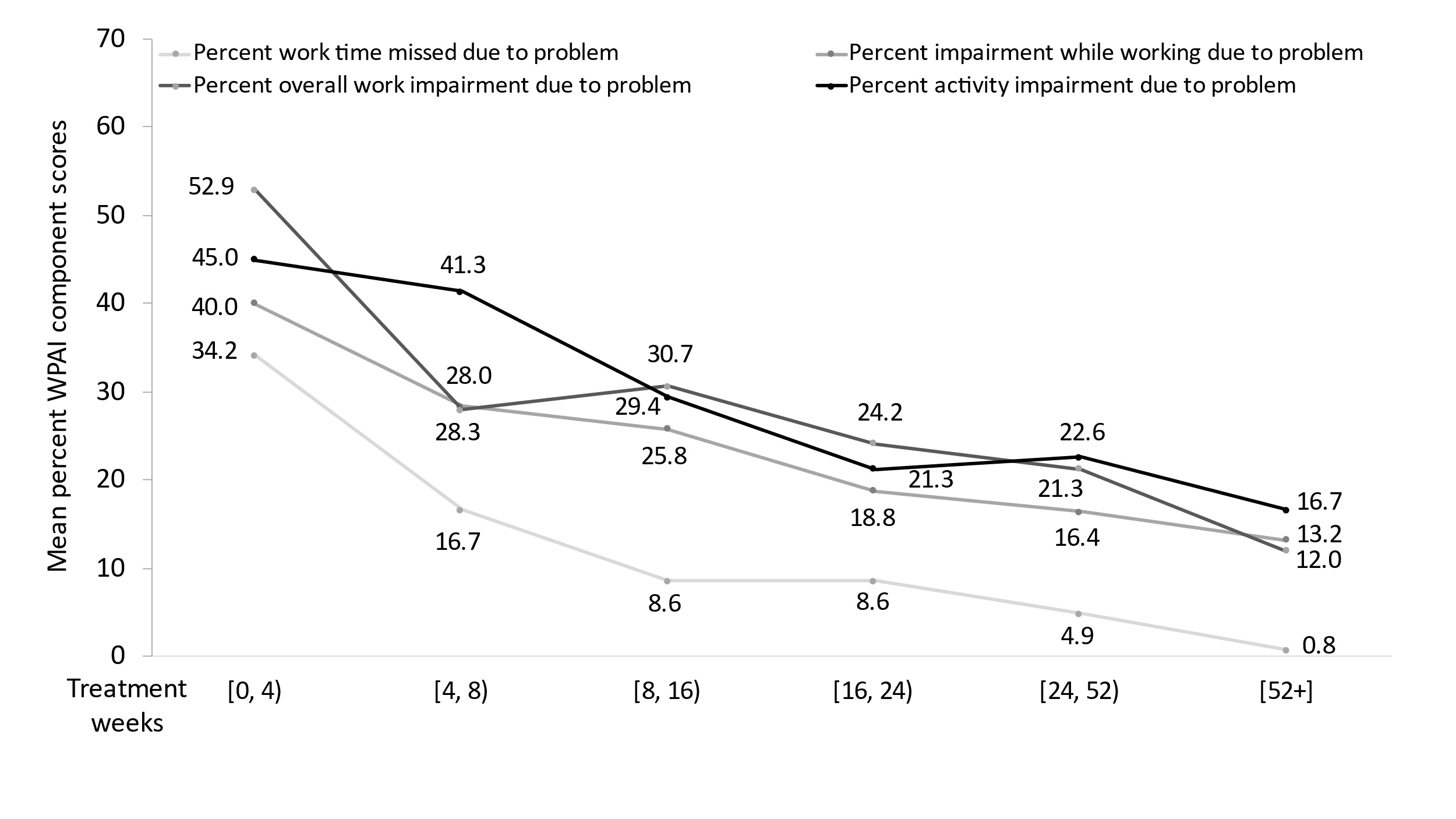


Base sizes for the four WPAI components varied. One observation per patient.

Linear regression with categorised time and additional covariates included.

The EQ-5D index total score ranges from <0.00 to 1.00, where higher scores indicate better HRQL; a 0.074-point change in the EQ-5D scale is considered a MCID.

The SIBDQ total score ranges from 10 indicating worst health to 70 indicating best health; a 9-point change in the SIBDQ is considered the MCID.

The WPAI component ranges from 0%, no impairment to 100%, total loss of work productivity or activity; a change of 6.5%, 6.1%, 7.3%, and 8.5% are considered to be MCIDs for absenteeism, presenteeism, overall work impairment, and total activity impairment, respectively.

EQ-5D-5L, EuroQol- 5 Dimension-5 Level; MCID, minimal clinically important difference; SE, standard error; SIBDQ, short version of the Inflammatory Bowel Disease Questionnaire; WPAI, Work Productivity and Activity Impairment.

UC, ulcerative colitis.
